# The impact of COVID-19 on mental health charity fundraising: An account from the perspective of fundraisers

**DOI:** 10.1192/j.eurpsy.2022.1786

**Published:** 2022-09-01

**Authors:** I. Bartolome, J. Beezhold, V. Selwyn, R. Gilmore, R. Howard, N. Henderson

**Affiliations:** 1 University of East Anglia, Norwich Medical School, Norwich, United Kingdom; 2 Hellesdon Hospital, Yare Ward, Norwich, United Kingdom

**Keywords:** mental health, Covid-19

## Abstract

**Introduction:**

The dawn of COVID-19 brought new rules, restrictions, and lockdowns but this led to the unlikely fall of many sectors, including the charitable sector. There has been a significant decline in funding received by mental health charities, especially during the pandemic. This study looks at the subsequent impact on fundraisers and mental health promotion during COVID-19.

**Objectives:**

The main aim was to uncover how the COVID-19 pandemic has affected the way that mental health charities fundraise, raise awareness, and promote mental health. This aimed to look at the impact of the COVID-19 pandemic on fundraisers supporting mental health charities and their opinion on whether fundraising has either helped or hindered mental health promotion.

**Methods:**

Accounts from fundraisers and local representatives for mental health charities during the COVID-19 pandemic were analysed. Common themes looked at the impact, adaptation, and reasons why fundraisers decided to help during a tough period for the charitable sector.

**Results:**

Mental health charities expressed difficulty in sourcing funds to support their users, especially in a vulnerable time. Fundraisers tried innovative ways to promote mental health and raise money for charities.

**Conclusions:**

Altruism and understanding how precious services such as mental health charities are for the population was the main driver for fundraisers. Mental health charities adapted by turning to online communication and reached out to fundraisers to continually highlight the importance of mental health to their users and fundraisers.

**Disclosure:**

No significant relationships.

